# Mast Cells in Alveolar Septa of COVID-19 Patients: A Pathogenic Pathway That May Link Interstitial Edema to Immunothrombosis

**DOI:** 10.3389/fimmu.2020.574862

**Published:** 2020-09-18

**Authors:** Jarbas da Silva Motta Junior, Anna Flavia Ribeiro dos Santos Miggiolaro, Seigo Nagashima, Caroline Busatta Vaz de Paula, Cristina Pellegrino Baena, Julio Scharfstein, Lucia de Noronha

**Affiliations:** ^1^School of Medicine, Pontifícia Universidade Católica do Paraná PUCPR, Curitiba, Brazil; ^2^Hospital Marcelino Champagnat, Curitiba, Brazil; ^3^Instituto de Biofísica Carlos Chagas Filho, Universidade Federal Do Rio de Janeiro, Rio de Janeiro, Brazil

**Keywords:** SARS-CoV-2, COVID 19, mast cells (MC), cell-mediated immunity, immune responses, interleukin-4 (IL-4)

## Abstract

It is currently believed that innate immunity is unable to prevent the spread of SARS-CoV-2 from the upper airways to the alveoli of high-risk groups of patients. SARS-CoV-2 replication in ACE-2-expressing pneumocytes can drive the diffuse alveolar injury through the cytokine storm and immunothrombosis by upregulating the transcription of chemokine/cytokines, unlike several other respiratory viruses. Here we report histopathology data obtained in post-mortem lung biopsies of COVID-19, showing the increased density of perivascular and septal mast cells (MCs) and IL-4-expressing cells (*n* = 6), in contrast to the numbers found in pandemic H1N1-induced pneumonia (*n* = 10) or Control specimens (*n* = 10). Noteworthy, COVID-19 lung biopsies showed a higher density of CD117^+^ cells, suggesting that c-kit positive MCs progenitors were recruited earlier to the alveolar septa. These findings suggest that MC proliferation/differentiation in the alveolar septa might be harnessed by the shift toward IL-4 expression in the inflamed alveolar septa. Future studies may clarify whether the fibrin-dependent generation of the hyaline membrane, processes that require the diffusion of procoagulative plasma factors into the alveolar lumen and the endothelial dysfunction, are preceded by MC-driven formation of interstitial edema in the alveolar septa.

## Introduction

As the pandemic caused by SARS-CoV-2 spreads to developing countries, the number of patients requiring intensive care treatment in overcrowded hospitals keeps increasing, limiting the clinical staff's capacity to prevent fatal outcomes. Viewed from the clinical perspective, the aims are to ameliorate respiratory functions by reducing the diffuse alveolar damage (DAD) that causes acute respiratory distress syndrome (ARDS) and to prevent the systemic thrombosis and multiple organ failure (1).

Although the temporal link between inflammatory edema and intra-alveolar hyaline membranes is yet to be proven, it has been established that infected pneumocytes undergoing apoptosis induce an inflammatory response conducting to interstitial edema and the consequent influx of plasma-borne procoagulative factor accumulating in interstitial spaces. The procoagulative factor-rich interstitial edema so would leak through intra-alveolar spaces triggering fibrin deposition over the damage alveolar septa (hyaline membranes). Converging with these infection-associated inflammatory lesions, patients afflicted with severe COVID-19 DAD mount a systemic immunothrombosis response that is steered by hyper-activated leukocytes. Particularly sensitive to microthrombi deposition, the alveolar capillaries are eventually clogged with thrombi, further disabling oxygen transfer to the bloodstream (2, 3).

Strategically localized in the subendothelial region, mast cells (MCs) are specialized innate sentinel cells that, upon activation, induce microvascular leakage, thereby linking immunity to pro-inflammatory and procoagulative networks as complement and the contact/kallikrein-kinin system (4–6). Despite evidence that MCs sense RNA viruses via TLR7 (7), MC-dependent increases in microvascular permeability might be potentiated by a myriad of soluble inflammatory mediators, including complement anaphylatoxins and tissue-derived alarmins (7, 8). Although MC targeting by cromolyn inevitably comes to mind as a low-cost therapeutic option, it has been reported that traditional MC stabilizers are far less potent inhibitors of MC release of inflammatory mediator than luteolin analogs, recently recognized as alternative drug therapy in corona-virus infection (8, 9).

The current study was partly motivated by a recent report showing the transcriptional profile of epithelial cells infected by SARS-Cov-2 vs. other human respiratory viruses (10). In contrast to the phenotypic properties of other viruses, the antagonism of (anti-viral) type-I and type III interferons are associated with a robust increase in the transcriptional program of chemokines/cytokines in SARS-Cov-2-infected epithelial cells. At first sight, these results raise the possibility that specific subsets of leukocytes (or their progenitors) might be recruited to the inflamed lung via upregulated secretion of specific chemokines. Although the dynamics of leukocyte recruitment to the alveolar septum of COVID-19 are unknown, we reasoned that some of the pathological features associated with severe COVID-19 could result from MC recruitment and/or maturation. Following this reasoning, we sought to compare the immunopathological aspects described in COVID-19 with knowledge already acquired in other respiratory viral pandemics, such as the pandemic Influenza A virus H1N1 subtype (H1N1pdm09) (11).

## Study Design and Post-Mortem Results

### Post-mortem Samples and Methods

The present study was approved by the National Research Ethics Committee (Conselho Nacional de Ética em Pesquisa—CONEP), protocol number 3.944.734/2020 (COVID-19 patients), and 2.550.445/2018 (H1N1pdm09 and Control patients). All methods were carried out following relevant guidelines and regulations. Families permitted the post-mortem biopsy of the cases of COVID-19 and H1N1pdm09.

We performed a histopathological study to compare the distribution of MCs in post-mortem lung biopsies of patients with COVID-19 (COVID-19 Group, *n* = 6, positive nasal swab RT-PCR for SARS-CoV-2 confirmed in more than one test) and post-mortem biopsies from patients with H1N1pdm09 infection (Group H1N1, *n* = 10, positive RT-PCR in fresh lung samples). Also included in this study was a Control group of lung samples (*n* = 10) from patients who died from neoplastic or cardiovascular diseases.

Clinical data were obtained from medical records during hospitalization in the Intensive Care Unit (ICU). A minimally invasive lung post-mortem biopsy was performed through a left anterior mini-thoracotomy with upper left lobe segment resection. The area's selection followed two criteria; (i) area with more severe lung injury identified on tomography and (ii) preferably in the left lung due to the mini-thoracotomy technique. The resected pieces were up to 3 × 3 cm.

The lung samples provided by post-mortem biopsy were formalin-fixed paraffin-embedded (FFPE) and stained with hematoxylin and eosin (H&E).

As read-outs, MCs and progenitors were identified by immunohistochemistry (IHQ) using a polyclonal antibody anti-CD117 (c-kit polyclonal rabbit anti-human, Dako Agilent, A4502) staining on FFPE samples. The secondary polymer was Reveal Polyvalent HRP-DAB Detection System, Spring Bioscience, CA, USA. Specificity controls were performed by (i) omitting the primary antibody (negative control) and (ii) testing skin tissues with mastocytosis (positive controls for anti-CD117). MC and degranulation responses were identifying by the toluidine blue (TB) stain.

CD117^+^ (only nucleated cells) and MCs were scored (IHQ and TB) exclusively in the alveolar septa and perivascular spaces, by counting the positive cells per high-power field (HPF −40× Olympus objective −0.26 mm^2^). Average scores were obtained by screening 10 randomized HPFs (total area of 2.6 mm^2^ per case).

The immunohistochemistry technique was also used to identify the expression of the interleukin-4 (anti-IL-4, Polyclonal/Rabbit, clone PA5-25165, dilution 1:200, Thermo Fisher). As stated for CD117 immunostaining, we used as secondary polymer Reveal Polyvalent HRP-DAB Detection System, Spring Bioscience, CA, USA, and positive and negative controls were performed.

The slides of IL-4 were scanned (Axio Scan Scanner. Z1, Carl Zeiss, Germany), and the ZEN software selected ten HPF (40× objective). The immunopositivity areas were measured by the Image-Pro Plus software version 4.5 (Media Cybernetics, USA). Subsequently, these areas were converted into percentages to enable statistical analysis.

The comparison of the quantitative variables of the two groups was performed using the non-parametric Kruskal Wallis test. Values of *p* < 0.05 indicated statistical significance. The data were analyzed using the IBM SPSS Statistics v.20.0 software. Armonk, NY: IBM Corp.

### Histopathological and Immunohistochemical Results

Clinical characteristics of the COVID-19, H1N1, and Control groups as age, survival (time from hospitalization to death), mechanical ventilation, tissue expression of IL-4, CD117^+^ cells, and MC score are listed in Table 1.

**Table 1:** Comparison between COVID-19, H1N1, and Control groups according to clinical and histopathological findings.

**Data**	**COVID-19 (*N* = 6)**	**H1N1 (*N* = 10)**	**CONTROL (*N* = 11)**
Gender	Male 4 (66.6%)	Male 8 (80.0%)	Male 8 (72.7%)
	Female 2 (33.4%)	Female 2 (20%)	Female 3 (27.3%)
		0.551^*^	0.793^#^
Age (years)^a^	76.5/80.5 (53–87)	43.5/44 (23–61)	42.3/45 (18–60)
		0.005^*^	0.003^#^
Comorbidities (number of cases)	Hypertension (4/6) Dyslipidemia (1/6) Hypothyroidism (1/6) Class II obesity (2/6) Dementia (2/6) Diabetes Mellitus (1/6) Chronic Kidney Disease (2/6) Coronary Disease (2/6)	**—**	**—**
Time from hospitalization to death (days)^a^	12.8/10 (2–32)	4.70/1.5 (1–19)	7.6/4 (1–46)
		0.006^*^	0.011^#^
Mechanical ventilation^a^	9.7/8 (0–21)	4.70/1.5 (1–19)	**—**
		0.185^*^	**—**
IL-4 tissue expression^a^^,^^b^	8.26/9.37 (0.71–13.39)	0.54/0.41 (0.19–1.12)	2.84/2.26 (0.23–7.41)
		0.003^*^	0.0509^#^
Number of CD117^+^ cells^a^^,^^c^	8.93/11.25 (2.90–12.70)	1.03/0.65 (0.20–3.10)	0.51/0.35 (0.10–1.40)
		0.002^*^	0.001^#^
Number of Mast cells (toluidine blue)^a^^,^^c^	1.58/1.35 (1.50–1.05)	0.09/0.10 (0.30–0.00)	0.05/0.00 (0.20–0.00)
		0.001^*^	0.001^#^

a *Average/Median (Min-Max)*.

b *Tissue expression in percentage per HPF*.

c *Number of CD117^+^/MCs in 10 HPF (average)*.

* *p-values obtained were compared between COVID-19 vs. H1N1*.

# *p-values obtained were compared between COVID-19 and Control group. p-values were performed using the non-parametric Mann-Whitney test (p < 0.05)*.

**Figure 1:**
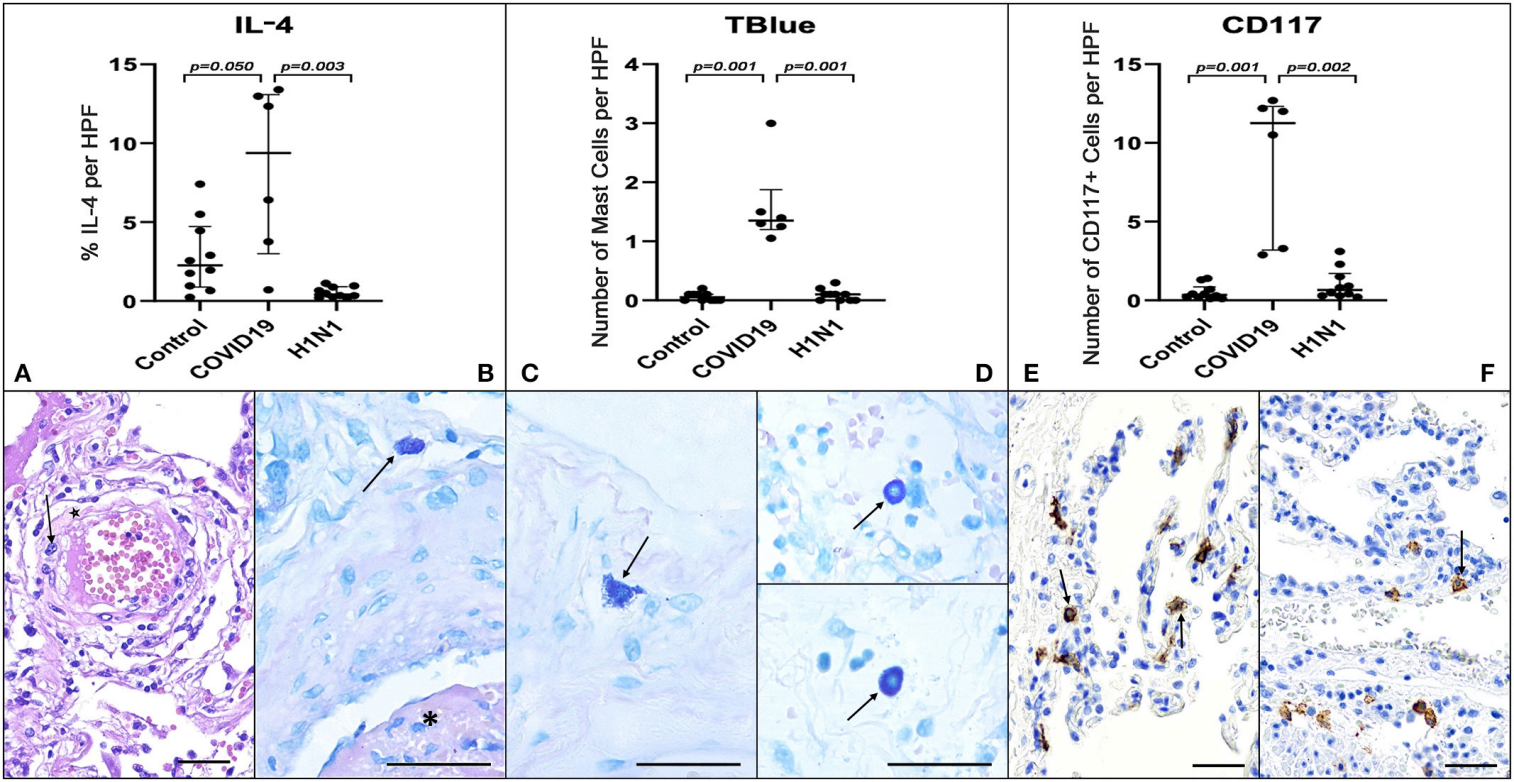
Graphs demonstrate the IL-4 tissue expression (percentage per HPF), the number of MCs (TB), and the number of CD117^+^ cells (IHQ) in the alveolar septa of the COVID-19 group compared to the H1N1 and Control groups. Non-parametric Kruskal Wallis test. Values of *p* < 0.05 indicated statistical significance. Histological section (H&E—**A**) shows evidence of microvascular involvement through endothelial activation (black arrow) and increased vascular permeability manifested by the formation of perivascular edema (asterisk). **(B)** (TB) shows thrombosis (asterisk) and perivascular MC (black arrow). **(C,D)** (TB) show evidence of degranulating (**C**—black arrow) and intact MCs (**D**—black arrows) and in the alveolar septa and perivascular spaces, in patients of COVID-19 **(C)**, H1N1 (**D**—upper) and Control (**D**—under) groups. **(E)** Of the COVID-19 group sample shows the massive presence of CD117^+^ nucleated cells (black arrows) compared to photomicrograph **(F)** of the H1N1 group sample (black arrow). Scale bar −50 μm. Scanned by the Axio Scan Scanner. Z1 (Carl Zeiss, Germany) with 40× **(A–D)** and 20× **(E,F)** objectives.

The COVID-19 group presented type 2 pneumocyte hyperplasia, hyaline membranes, and septal thickness with mild lymphocytic infiltration characterizing proliferative DAD. Furthermore, COVID-19 biopsies showed numerous fibrinous thrombi (Figure 1) following by neutrophilic endotheliitis. Signs of secondary bacterial pneumonia were not observed. Although the formation of hyaline membranes was also observed in H1N1 biopsies, the histopathological features of these patients were distinguished by a marked increase in septal thickness associated with lymphocytic infiltration and a massive intra-alveolar influx of neutrophils. There was no expressive neutrophilic endotheliitis in H1N1 cases. Signs of bacterial coinfection were found in 8 cases.

COVID-19 group samples presented numerous MCs. These innate sentinel cells were more frequently localized in the perivascular spaces between the alveolar sacs and terminal bronchioles and in the alveolar septa, close to the alveolar capillaries. The results presented in Figure 1 and Table 1 show the distribution of CD117^+^ cells (IHQ) and MCs (TB) in post-mortem lung biopsies of patients with COVID-19 with a striking difference in the number of CD117^+^ cells and MCs between the COVID-19 group and the H1N1 (*p* = 0.002 and 0.001, respectively) and Control (*p* = 0.001 and 0.001, respectively) groups. The number of MCs counted by the TB technique was about 5-fold lower than the number of CD117^+^ cells (IHC). MC degranulation (TB) was consistently seen in the alveolar septa of COVID-19, as well as TB^+^ individual granules (non-nucleated) dispersed in tissues and MCs with depleted cytoplasm (Supplementary Figure 1).

By morphological criteria, the pulmonary cells expressing IL-4 are mainly alveolar macrophages and type II pneumocytes in all three groups (Supplementary Figure 1). The COVID-19 group presents statistically significant higher tissue expression of IL-4 (Table 1 and Figure 1) compared to H1N1 (*p* = 0.003) and Control groups (*p* = 0.0509, borderline).

## Discussion

Despite their functional heterogeneity, human MCs are known to produce a wide range of pro-inflammatory molecules, including distinct categories of mediators stored in secretory granules. Among those that modulate endothelial barrier function are classical vasoactive mediators, such as histamine, leukotriene B4 and LTC4, prostaglandin D2, vascular endothelial growth factor, serine proteases, such as tryptase and chymase. MCs also contribute to cytokine networking by releasing the type-2 cytokine IL-4 and IL-6, a pivotal player in the systemic cytokine storm associated with severe COVID-19 (12).

Although MC-derived histamine is a classical inducer of microvascular leakage, previous analysis of MC function in allergic lung disease (13) linked bradykinin-induced inflammation to extravascular activation of plasma-borne contact factors by heparin (14) and/or polyphosphates (15), both of which are released from MC secretory granules. We observed the presence of degranulated MCs in the alveolar septa, regardless of the nature of the inflammatory mediators released by infected epithelial cells. It is tempting to speculate that MC-driven microvascular leakage favors the intra-alveolar formation of the hyaline membrane by harnessing the diffusion of plasma procoagulative factors from the interstitial space into the alveolar sac.

Previous studies in mice infected by SARS-Cov supported the concept that ACE-2 (angiotensin-converting enzyme 2) internalization following virus entry in epithelial cells worsens lung inflammation by down-modulating the surface expression of ACE-2, a key regulator of the renin-angiotensin system (16). Although the anti-inflammatory effects of ACE-2 are usually attributed to its ability to degrade angiotensin II, studies in mice deficient of ACE-2 demonstrated that this metalloprotease dampens LPS-induced inflammation in the lung by degrading des-Arg-bradykinin, i.e., the high-affinity ligand of B1R, a subtype of endothelial bradykinin receptors whose surface expression is strongly upregulated by pro-inflammatory cytokines (16, 17).

Multiple mechanisms, including complement components (e.g., C5a) (18) and inflammatory cytokines (e.g., TNF-alpha), can mediate MC activation. Among these, immunoglobulin E (IgE) is probably the best known. Regarding the endothelial injury triggering immunothrombosis, activated MCs can release pro-inflammatory cytokines (e.g., IL-6) to induce matrix-degrading protease expression from endothelial cells, causing endothelial barrier injury. Activated MCs can also release proteases (e.g., tryptase) to induce vascular cell apoptosis, angiogenic factors to stimulate angiogenesis and histamine (19).

Another contrasting histopathological feature between COVID-19 and H1N1 described in our report was the increased numbers of IL4-expressing cells in the alveolar septa of patients with severe COVID 19. Classically produced by Th2 lymphocytes, IL-4 is a type-2 cytokine also secreted by MC, basophils, eosinophils, and innate lymphoid cells-2. Interestingly, longitudinal analysis of peripheral immune profiles of SARS-Cov-2 patients has recently revealed that cytokine signatures displayed by severe COVID-19 patients are shifted toward the type-2 cytokine profile. At the same time, type-1/3 responses are shared with the profiles of patients with moderate clinical manifestations (20). Although peripheral IL-4 responses were not as prominent as IL-5, IL-13, IgE, and eosinophils, the authors pointed out that severe clinical outcomes were associated with an upward trend for the IL-4 cytokine. Intriguingly, they also reported that levels of IgE levels were significantly higher in severe patients and continued to increase as the disease progressed.

IL-4 can impair endothelial barrier function by inducing cytoskeleton remodeling. IL-4 may upregulate the expression of vascular cell adhesion molecule-1 (VCAM-1) and monocyte chemotactic protein-1 (MCP-1), induce hyperpermeability and cause microvascular leakage (21). By morphological criteria, IL-4-expressing cells in COVID-19 patients include alveolar M2 macrophages and type II pneumocytes. It is unclear whether a fraction of the IL4-expressing cells found in the alveolar septa are represented in the single-cell RNA sequencing profiles generated from peripheral blood mononuclear cells of COVID-19 patients. Interestingly, there is a precedent that IL-4 can induce hyperpermeability of vascular endothelial cells through the activation of the Wnt5A signaling pathway (21).

The presence of mast cells at the site in human tissues associated with its ability to release proangiogenic VEGF-A, histamine, tumor necrosis factor-α and several other vasoactive mediators of endothelial activation in the inflamed alveolar septa may have an indirect impact on platelet adhesion to the endothelial lining, and subsequent fibrin formation via cooperative activation of extrinsic/intrinsic pathways of coagulation (14, 15, 22). Inevitably, under the adverse influence of the cytokine storm, the risk of the microthrombi formation may increase. Besides, SARS-CoV-2 infection can induce lung tissue damage, resulting in activation, aggregation, and entrapment of the platelet leading to thrombosis and consumption coagulopathy (23).

While supporting the notion drug targeting of the KKS/B2R/B1R axis may protect patients from SARS (24), the experimental findings and arguments outlined in this study should encourage clinicians to conduct randomized trials to evaluate the potential benefit of low-cost therapy with MC stabilizers. Another potentially useful strategy could be to target the subsets of chemokines that presumably recruit C-kit positive MC progenitors to the injured/infected alveolar tissue, or by blocking the activity of mediators and growth factors drive MC maturation in the alveolar septa (25–27). This is the first report in the literature showing that increased MC density is a distinguishing pathological feature in the lungs of COVID-19 patients compared to HIN1-induced pneumonia.

The contrasting scores of CD117^+^ cells and MC numbers (TB) in the alveolar septa of COVID-19 vs. H1N1 groups might reflect different rates of progenitors to the lung. Since TB staining is dependent on the presence of granules in the cytoplasm of tissue MCs when activated, MC may be undergoing active degranulation and showing reduced granule proteins in their cytoplasm, which in turn would reduce TB staining. Hence tissue MC degranulation could explain a discrepancy between CD117 and TB staining in the alveolar septa. Additional clinical studies are required to determine whether these discrepant phenotypes are due to age factors or a more extended time from hospitalization to death of COVID-19 patients (mean 12.8 days) than H1N1 patients (mean 4.7 days). Finally, the finding that CD117^+^ cells showed higher scores (about 5-fold over TB) suggests that the c-kit positive cells could include MCs and their progenitors, blood progenitor cells, CD31^+^ cells, and type 2 innate lymphoid cells (28).

Our study presents some limitations that merit consideration. Given the scarcity of larger samples of collected post-mortem lung biopsies in this highly contagious environment of COVID-19 Intensive Care Units, our sample is limited. It is essential to interpret our findings with caution and validate them in other samples to replicate our results. Additionally, data based on FFPE post-mortem samples only provide static information at the time of death. They cannot reconstruct the evolving disease process. Furthermore, the COVID-19 and H1N1pdm09 are different pandemic diseases concerning their demographic risk groups, pathophysiology mechanisms, and coinfection prevalence.

In conclusion, we propose that drugs that uncouple the MC link to pro-inflammatory proteolytic networks, such as complement and the kallikrein-kinin cascade, may inhibit interstitial edema in the alveolar septa. By protecting the microvasculature from excessive leakage, MC stabilizing drugs may limit the intra-alveolar formation of the hyaline membrane while attenuating immunothrombosis in severe COVID-19.

## Data Availability Statement

All datasets generated for this study are included in the article/Supplementary Material.

## Ethics Statement

The studies involving human participants were reviewed and approved by National Research Ethics Committee (Conselho Nacional de Ética em Pesquisa—CONEP), protocol number 3.944.734/2020 and 2.550.445/2018. The patients/participants provided their written informed consent to participate in this study.

## Author Contributions

JM, AM, CB, JS, and LN: study design, data collection, writing, and critically reviewing the manuscript. JM, AM, SN, CP, and LN: data analysis and interpretation. All authors contributed to the article and approved the submitted version.

## Conflict of Interest

The authors declare that the research was conducted in the absence of any commercial or financial relationships that could be construed as a potential conflict of interest.

## Supplementary Material

Supplementary Figure 1 Photomicrographs of the COVID-19 group samples (C1–C6) showing IL-4 tissue expression mainly in pneumocytes (asterisks), CD117^+^ nucleated cells (black arrows), degranulating (black arrow), and depleted (dashed arrow) MCs and some free granules dispersed (arrowhead) in the alveolar septa and perivascular spaces.
