# Supplementary figures and images for: Mast Cells in Alveolar Septa of COVID-19 Patients: A Pathogenic Pathway That May Link Interstitial Edema to Immunothrombosis
Source: Front Immunol. 2020 Sep 18;11:574862. doi: 10.3389/fimmu.2020.574862 (PMC7530169; doi:10.3389/fimmu.2020.574862)

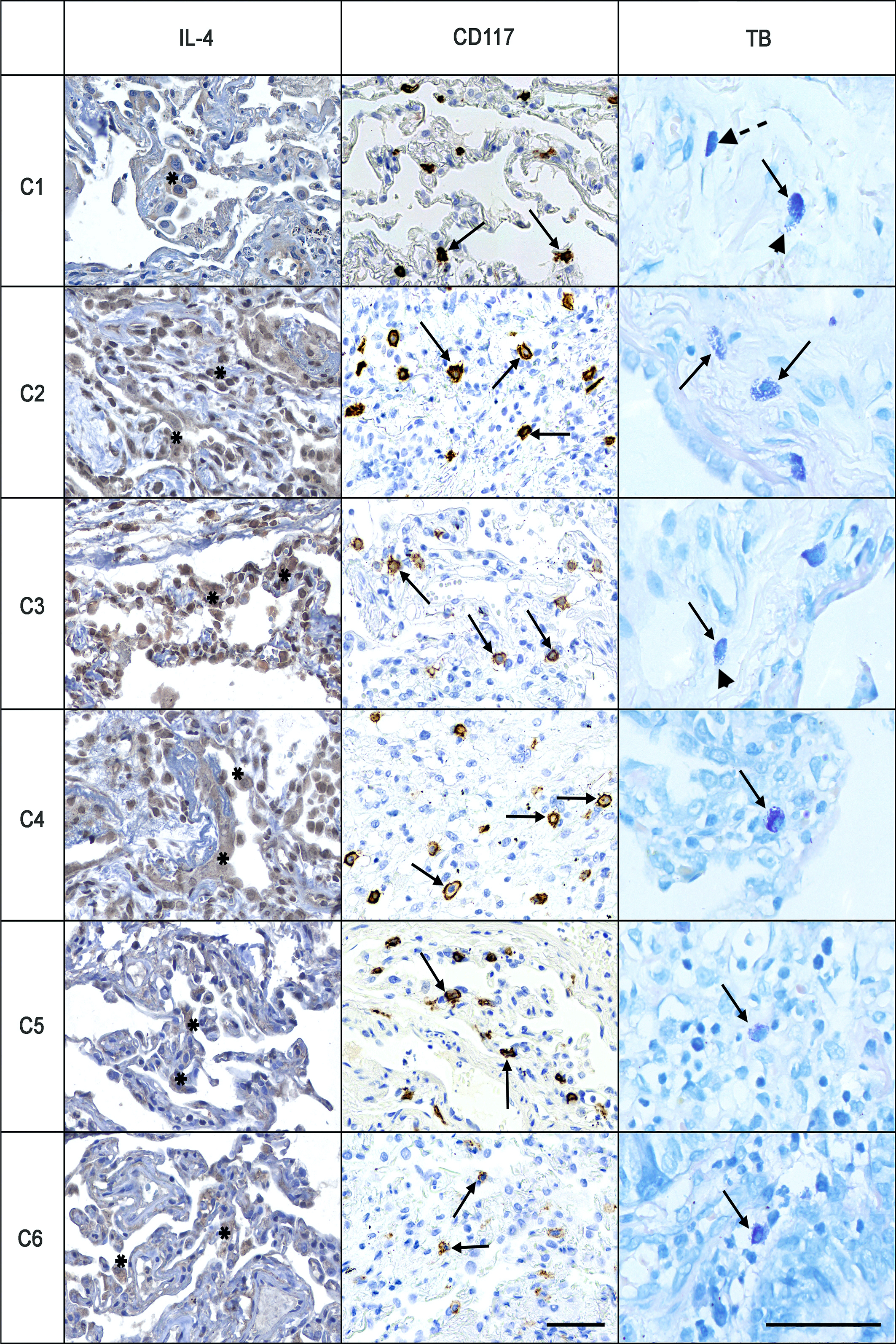

Supplement: Supplementary Figure 1 — Photomicrographs of the COVID-19 group samples (C1–C6) showing IL-4 tissue expression mainly in pneumocytes (asterisks), CD117+ nucleated cells (black arrows), degranulating (black arrow), and depleted (dashed arrow) MCs and some free granules dispersed (arrowhead) in the alveolar septa and perivascular spaces. [file Image_1.JPEG]
